# Characterizing the effects of different chemicals on stem bending of cut snapdragon flower

**DOI:** 10.1186/s13007-021-00835-1

**Published:** 2022-01-13

**Authors:** May Thu Soe, Aung Htay Naing, Soo Rin Kim, Chang Kil Kim

**Affiliations:** 1 Department of Horticulture, Kyungpook National University, Daegu, 41566 South Korea; 2 School of Food Science and Biotechnology, Kyungpook National University, Daegu, 41566 South Korea

**Keywords:** Ethylene production, Gene expression, Growth supplements, Lignin content, Petal senescence

## Abstract

**Background:**

This study investigated the effects of ethylene release compounds (ethephon), ethylene-action inhibitors (silver thiosulfate: STS), and nitric oxide donor (sodium nitroprusside: SNP) on stem bending of snapdragon flowers. Moreover, the effects of plant growth supplements [6-benzyladenine (BA), gibberellic acid 3 (GA_3_), and calcium chloride (CaCl_2_)] on the stem bending were also extensively investigated.

**Results:**

Ethephon completely prevented stem bending until 9 days after treatment (9 DAT). STS exhibited the highest bending rate, while SNP did not significantly affect the bending compared to the controls. The bending results were associated with the results of stem curvature, relative shoot elongation, ethylene production, and lignin content, that are involved in the stem bending mechanism. This was proven by the expression analysis of genes involved in ethylene and lignin biosynthetic pathways. The addition of plant growth supplements slightly or significantly delayed stem bending in the treatments (control, SNP, and STS) and significantly reduced petal senescence in ethephon at 9 DAT.

**Conclusion:**

These results show the preventive role of ethephon in the stem bending of cut snapdragon. Moreover, the combination of ethephon with supplements also provided information that could guide the development of strategies to delay stem bending in other cut flowers that undergo serious bending during a short vase life.

**Supplementary Information:**

The online version contains supplementary material available at 10.1186/s13007-021-00835-1.

## Introduction

Snapdragon (*Antirrhinum majus* L.) is widely used as a cut flower in the floricultural industry due to its diverse and attractive colors [1]. However, the occurrence of stem bending during a short vase life period is a major postharvest problem of cut snapdragon flowers, which threatens flower growers and users. Generally, stem bending of cut flowers is associated with a poor lignification mechanism during stem elongation [2–4]; the reduction of lignin content weakens the rigidity of the stem and the vascular tissue which supports the water and minerals in the xylem, thus reducing the mechanical resistance of the whole flowers as well as disrupting water and mineral transport to the flowers and leading to stem bending.

Efforts have been made to overcome poor lignification mechanisms and therefore improve stem bending using ethylene-action inhibitors, such as silver thiosulfate (STS) and 1-methylcyclopropene (1-MCP) [5–8]. Celikel et al. [5] and Woltering et al. [8] claimed that STS or 1-MCP did not significantly affect the stem bending of snapdragon, whereas Philosoph-Hadas et al. [6, 7] stated that STS can block the gravitropic response of flowers. Conversely, the ethylene-releasing compound 2-chloroethylphosphonic acid (Ethephon) has been shown to inhibit stem elongation, which accelerates the stem bending of cut flowers [2, 9–11], and its application in vase life solution delayed stem bending of cut tulip and snapdragon flowers [2, 11]. Ethephon has been shown to have promoting effects on lignification [2, 12, 13]. Delaying of stem bending by sodium nitroprusside (SNP) has been reported in gerbera and *Calendula officinalis* L. [3, 14, 15], whereas SNP promoted lignification and delayed the stem bending in cut gerbera [3]. Although ethephon has a promoting effect on lignification and preventive effect on stem bending [2, 11–13], as described above, the ethylene inhibitors STS or 1-MCP blocked the gravitropic bending of snapdragon [6, 7]. In addition, the effect of SNP on stem bendingylene-induced changes in lignificatio in snapdragon has not been investigated. Therefore, it is interesting to investigate how these chemicals (ethephon, STS, and SNP) affect the stem bending of the cut snapdragon.

van Doorn et al. [11] and Naing et al. [2] observed that ethephon rapidly enhanced petal senescence due to overproduction of ethylene. van Doorn et al. [11] revealed the negative effect of ethylene on petal senescence by adding the supplements gibberellic acid (GA_3_), benzyl adenine (BA), and calcium chloride (CaCl_2_) to the vase solution, however, Naing et al. [2] did not test the effects of the supplement on the reduction of petal senescence in cut snapdragon. Therefore, the combined effects of the treatments (ethephon, STS, and SNP) with supplements on stem bending and petal senescence of snapdragon should be investigated.

In this study, we treated snapdragon cut flowers with ethephon, STS, and SNP and investigated their roles in the stem bending of the cut snapdragon by assessing several factors associated with stem bending. Moreover, the effects of supplements on stem bending and petal senescence were also investigated.

## Results

### Stem curvature and bending

The degree of stem curvature observed in the control treatment was significantly higher than those of the ethephon, SNP, and STS treatments at 3 days after treatment (DAT); the stem curvatures of the treatments were in the order STS > SNP > ethephon. At 6 DAT the curvature degrees had increased in all treatments except ethephon and the curvature induced by STS had overtaken that of the control (STS > control > SNP). Significant increases in the degrees were observed at 9 DAT, particularly for STS and SNP, however the curvatures of the control and SNP were not significantly different. Surprisingly, the increment was not observed in ethephon. The addition of supplements (BA, GA_3_, and CaCl_2_) to the control, STS, and SNP significantly reduced the degrees of curvature at 3, 6, and 9 DAT. However, the supplements did not significantly impact the effect of ethephon on the curvature (Table 1).

**Table 1:** Effect of ethephon, STS, and SNP alone or in combination with supplements on stimulation of curvature degree of stems at 3, 6, and 9 days after treatment

Treatment	Curvature (degree)		
	3D	6D	9D
Control	33 ± 7 a	48 ± 4 c	49 ± 4 c
STS	30 ± 9 b	72 ± 4 a	148 ± 6 a
SNP	26.5 ± 4 c	35 ± 4 d	46 ± 4 c
Ethephon	20 ± 2 d	22 ± 4 e	22 ± 4 e
Control + (BA, GA_3_, CaCl_2_)	20 ± 2 d	23 ± 4 e	27 ± 4 d
STS + (BA, GA_3_, CaCl_2_)	28.3 ± 3 c	58 ± 4 b	101 ± 5 b
SNP + (BA, GA_3_, CaCl_2_)	15 ± 4 e	22 ± 3 e	23 ± 5 e
Ethephon + (BA, GA_3_, CaCl_2_)	22 ± 3 d	23 ± 2 e	23 ± 2 e

Data represent the mean of three replicates, and error bars indicate the standard error. Means with the same letters are not significantly different according to the least significant difference test (LSDT, p < 0.05)

**Fig. 1:**
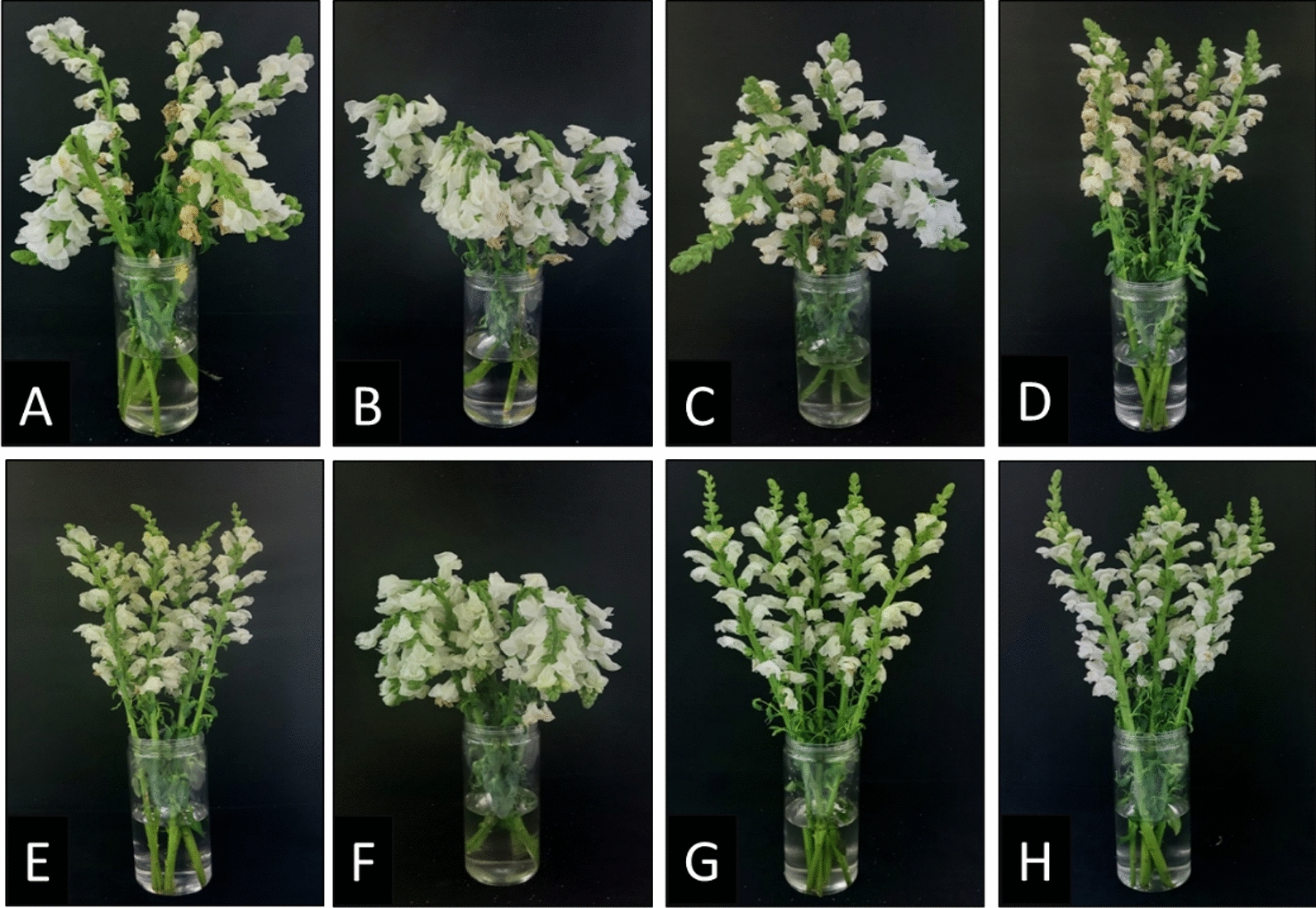
Comparison of the effects of SNP, STS, ethephon, and control alone or in combination with supplements on the improvement of postharvest quality of snapdragon cut flowers. **A** Control, **B** STS, **C** SNP, **D** Ethephon, **E** Control + (BA, GA_3_, CaCl_2_), **F** STS + (BA, GA_3_, CaCl_2_), **G** SNP + (BA, GA3, CaCl2), **H** Ethephon + (BA, GA3, CaCl2). The photos were taken at 9 DAT

No stem bending (stem with an angle of more than 45°) was observed at 3 DAT. At 6 DAT, about 50% of the flowers from the control and STS and 20% of SNP-treated flowers were bent. At 9 DAT, all flowers from STS were bent, while 65% and 50% of the flowers from the control and SNP treatments showed bending, respectively. Surprisingly, no stem bending was observed in the flowers treated with ethephon even at 9 days (Fig. 1A–H). In addition, adding the supplements to the treatments significantly delayed the stem bending of flowers, except for ethephon (Table 2).

**Table 2:** Effect of ethephon, STS, and SNP alone or in combination with supplements on stem bending at 3, 6, and 9 days after treatment

Treatment	Bent stem (% of total)		
	3D	6D	9D
Control	0	50 b	65 c
STS	0	55 a	100 a
SNP	0	20 c	50 c
Ethephon	0	0 d	0 e
Control + (BA, GA_3_, CaCl_2_)	0	0 d	30 d
STS + (BA, GA_3_, CaCl_2_)	0	40 b	88 b
SNP + (BA, GA_3_, CaCl_2_)	0	5 d	20 d
Ethephon + (BA, GA_3_, CaCl_2_)	0	0 d	0e

Data represent the mean of three replicates, and error bars indicate the standard error. Means with the same letters are not significantly different according to the least significant difference test (LSDT, p < 0.05)

### Relative shoot elongation (RSE)

**Fig. 2:**
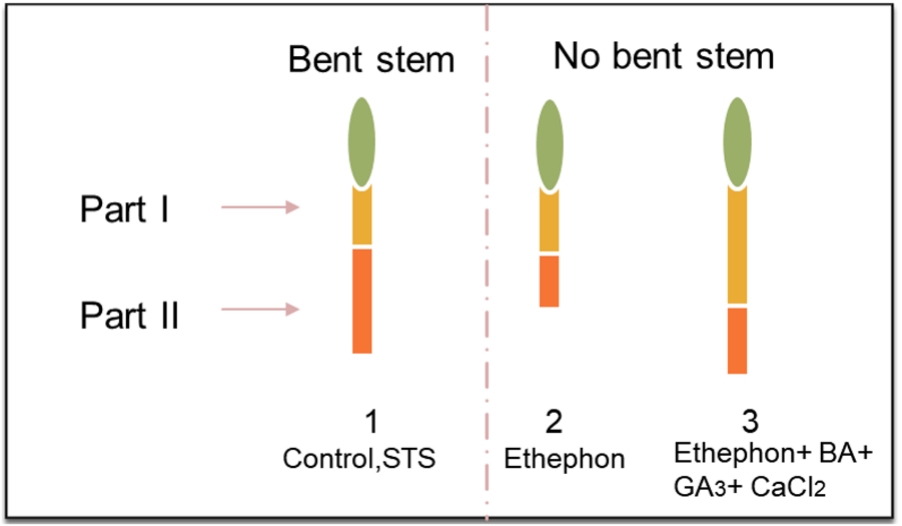
Comparison of shoot elongation of part I and part II between bent stem and no bent stem

The RSE observed in the different zones differed significantly. Among the three zones, the RSE of part II was the highest, followed by part I and part III. The RSE was not significantly different in part I for all treatments, while the RSE of part II was observed in the order STS and control > SNP > ethephon (Table 3). In part III, ethephon did not completely stimulate stem elongation, while those stimulated by control and SNP were the same and significantly lower than that stimulated by STS. The addition of supplements to the treatments significantly or slightly stimulated RSE depending on the treatments. Based on these results, the RSE results observed in part II were more likely to be associated with a higher bending rate than those of RSE in part I. This was further proved by comparing the RSE results of parts I and II between the bending and unbending stems (Fig. 2).

**Table 3:** Effect of ethephon, STS, and SNP alone or in combination with supplements on relative shoot elongation of shoots at 6 days after treatment

Treatment	Relative shoot growth (% of initial)		
	Part I	Part II	Part III
Control	18 ± 2.3 c	24 ± 2 b	2 ± 0.6 b
STS	14 ± 1.2 c	26 ± 1.5 ab	4 ± 0.6 a
SNP	14 ± 0.6 c	18 ± 1.7 c	2 ± 0.5 b
Ethephon	16 ± 0.6 c	12 ± 0.6 d	0 c
Control + (BA, GA_3_, CaCl_2_)	34 ± 2.3 b	24 ± 1.5 b	4 ± 0.3 a
STS + (BA, GA_3_, CaCl_2_)	29 ± 1.5 b	27 ± 1.5 a	4 ± 0.3 a
SNP + (BA, GA_3_, CaCl_2_)	32 ± 1.2 b	24 ± 1.2 b	2 ± 0.3 b
Ethephon + (BA, GA_3_, CaCl_2_)	42 ± 1.5 a	12 ± 1 d	2 ± 0.5 b

Data represent the mean of three replicates, and error bars indicate the standard error. Means with the same letters are not significantly different according to the least significant difference test (LSDT, p < 0.05)

### Lignin content and ethylene production

Among all treatments (control, STS, SNP, and ethephon), the lignin content obtained from the ethephon-treated stems was the highest throughout the vase life period. However, the lignin contents of the stems obtained from control, SNP, and STS treatments were not significantly different, despite the presence of different lignin contents in the treatments (SNP > control > STS) (Table 4). Continuous reduction of the lignin content was observed in all treatments along with increasing vase life periods, but the addition of the supplements to the treatments promoted lignin content in all treatments, except for ethephon, throughout the vase life period. Ethylene production in the stem sections of the control, ethephon, and ST, which were excised from the putative bending zone (about 100–150 mm distance from the apex of the shoot), was assessed at 6 DAT. The ethylene production in the stems was observed to be the highest in ethephon followed by the control and STS (Table 5).

**Table 4:** Effect of ethephon, STS, and SNP alone or in combination with supplements on maintenance of lignin content from the bending zone of the stems at 3, 6, and 9 days after treatment

Treatment	Lignin content (A280 mg^-1^ protein)		
	3D	6D	9D
Control	1.2 ± 0.1 cd	0.4 ± 0.09 c	0.16 ± 0.05 b
STS	1 ± 0.17 d	0.33 ± 0.01 c	0.14 ± 0.01 b
SNP	1.5 ± 0.29 bc	0.45 ± 0.1 c	0.18 ± 0.01 b
Ethephon	2.5 ± 0.29 a	1.25 ± 0.14ab	0.55 ± 0.12 a
Control + (BA, GA_3_, CaCl_2_)	1.9 ± 0.12 b	1.34 ± 0.16a	0.45 ± 0.05a
STS + (BA, GA_3_, CaCl_2_)	1.5 ± 0.17 bc	0.42 ± 0.08 c	0.27 ± 0.02 b
SNP + (BA, GA_3_, CaCl_2_)	1.8 ± 0.15 bc	0.99 ± 0.08 b	0.5 ± 0.06 a
Ethephon + (BA, GA_3_, CaCl_2_)	2.1 ± 0.1 ab	1.09 ± 0.04ab	0.62 ± 0.08 a

Data represent the mean of three replicates, and error bars indicate the standard error. Means with the same letters are not significantly different according to the least significant difference test (LSDT, p < 0.05)

**Table 5:** Effect of ethephon, STS, and control on ethylene production from bending zones of the stems at 6 days after treatment

Treatment	Ethylene production (nl/g/h)
Control	4.4 ± 0.2 b
STS	2.6 ± 0.9 c
Ethephon	11.6 ± 0.2 a

Data represent the mean of three replicates, and error bars indicate the standard error. Means with the same letters are not significantly different according to the least significant difference test (LSDT, p < 0.05)

### Expression of lignin and ethylene biosynthesis genes

**Fig. 3:**
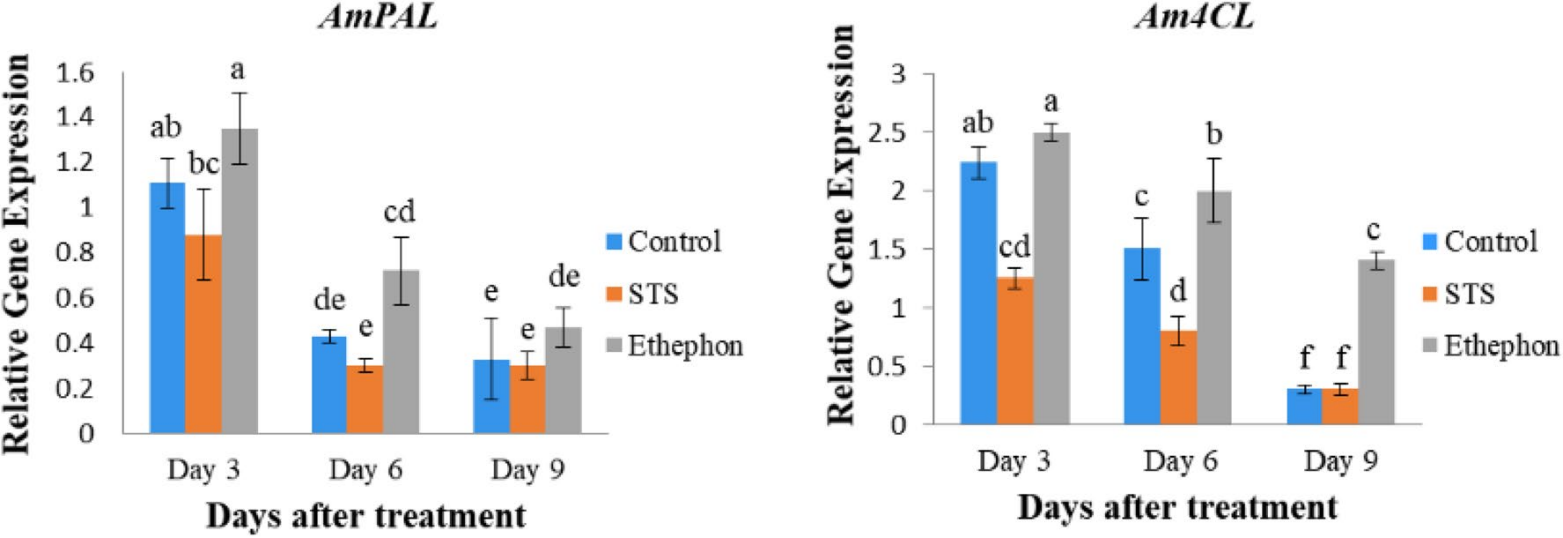
Comparison of the transcript levels of lignin biosynthesis genes in the bending zones of the stems treated with STS, ethephon, and control at 3, 6, and 9 days after treatment Data represent the means of three replicates, and error bars indicate standard error. Means with the same letters are not significantly different by Lest Significant Different Test (LSDT, p < 0.05)

**Fig. 4:**
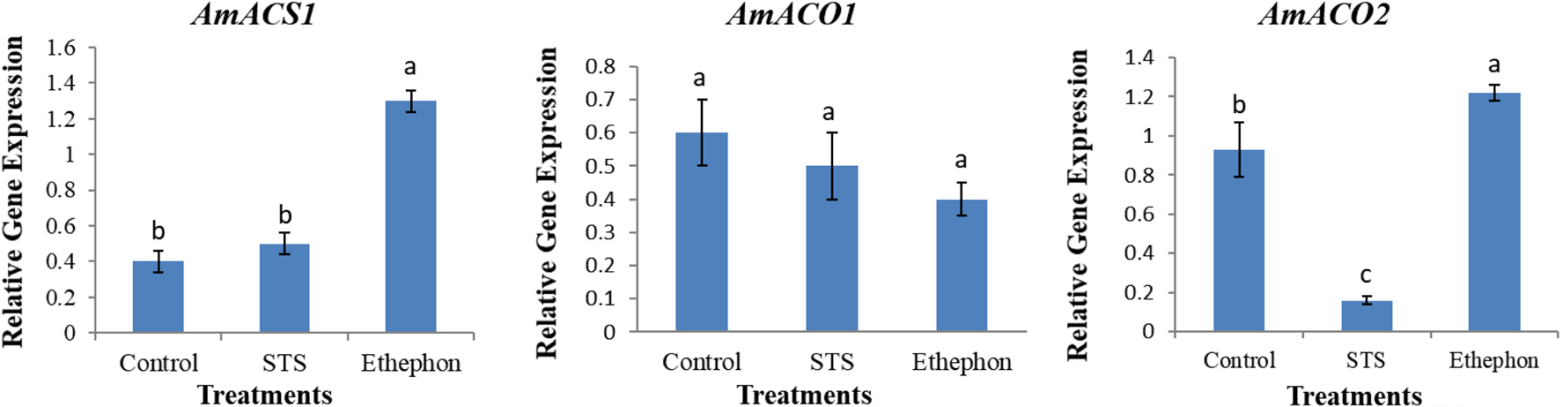
Comparison of the transcript levels of ethylene biosynthesis genes in the bending zones of the stems treated with STS, ethephon, and control at 6 days after treatment Data represent the means of three replicates, and error bars indicate standard error. Means with the same letters are not significantly different by Lest Significant Different Test (LSDT, p < 0.05)

The expression levels of phenylalanine ammonia-lyase (*PAL*) and 4-coumarate, CoA ligase (*4CL*) genes involved in lignin biosynthesis, were highest in all treatments at 3 DAT, followed by 6 DAT and 9 DAT, indicating a continuous reduction in gene expression with increasing vase life period (Fig. 3). However, their expression levels were higher in the ethephon than the control and STS throughout the vase life; the expression levels in the control were higher than those in the STS (except at 9 DAT). These results confirm that the lignin contents in the treatments were generally consistent with the expression levels of the lignin biosynthetic genes. Expression levels of ethylene biosynthesis genes, such as 1-aminocyclopropane-1-carboxylic acid (ACC) synthetase 1 (*ACS1*), ACC oxidase (*ACO1*), and ACC oxidase 2 (*ACO2*), in the stems were also assessed to reveal the association between their expression and ethylene production. The expression level of *ACS1* was significantly higher in the ethephon than in the control and STS, despite no significant differences of expression between the control and STS. In contrast to *ACS1*, ACO*1* expression levels were not significantly different among the treatments, but *ACO2* expression in the ethephon was observed to be the highest, followed by the control and STS (Fig. 4). Ethylene production was more likely associated with the expression levels of *ACO2,* rather than those of *ACS1* and *ACO1*.

### The petal senescence

When assessing how petal senescence was affected by the treatments, no senescence was observed in the control, SNP, or STS at 3 DAT; however, 20% of the flowers treated with ethephon exhibited senescence. At 6 DAT, senescence occurred in all treatments. The order of the senescence rate was as follows: ethephon > control > SNP > STS. When the vase life period was extended to 9 days, 90% of the flowers treated with ethephon showed petal wilting and browning at the corner in half of the flower spikes, compared to 60% in the control and SNP treatments (Table 4). Overall, compared to the control, STS significantly reduced petal senescence and ethephon rapidly accelerated senescence. However, no significant effect on senescence was observed between the SNP and control at 6 and 9 DAT. The addition of the supplements (BA, GA_3_, and CaCl_2_) to the control, STS, and SNP treatments did not significantly affect petal senescence; however, the addition of the supplements significantly reduced senescence (from 90 to 50%) in the flowers treated with ethephon at 9 DAT (Table 6).

**Table 6:** Effect of ethephon, STS, and SNP alone or in combination with supplements on petal senescence at 3, 6, and 9 days after treatment

Treatment	Petal senescence (% of total)		
	3D	6D	9D
Control	0 b	40 ± 4 ab	60 ± 5.8 b
STS	0 b	25 ± 2.9 b	40 ± 5.8 c
SNP	0 b	30 ± 2.9 b	60 ± 2.9 b
Ethephon	20 ± 4 a	50 ± 7.6 a	90 ± 4 a
Control + (BA, GA_3_, CaCl_2_)	0 b	25 ± 5 b	50 ± 8.7 bc
STS + (BA, GA_3_, CaCl_2_)	0 b	30 ± 2.9 ab	40 ± 2.9 c
SNP + (BA, GA_3_, CaCl_2_)	0 b	35 ± 5 ab	50 ± 5 bc
Ethephon + (BA, GA_3_, CaCl_2_)	0 b	35 ± 3 ab	50 ± 2.9 bc

Data represent the mean of three replicates, and error bars indicate the standard error. Means with the same letters are not significantly different according to the least significant difference test (LSDT, p < 0.05)

## Discussion

In this study, we investigated the effects of ethephon, STS, and SNP individually or in combination with supplements on the stem bending of snapdragon by assessing several factors, such as stem curvature, stem bending rate, RSE, lignin content, and ethylene production, and their related gene expression. In addition, their effects on petal senescence were assessed.

The curvature degree results were significantly correlated with the stem bending rates throughout the vase life period for all treatments. In addition, at 9 DAT, the lack of significant difference in the bending rate between the control and SNP was also associated with no significant difference of their curvatures. We found that SNP did not effectively inhibit curvature when the vase life period was extended to 9 days. However, SNP has been reported to delay stem bending in gerberas and *Calendula officinalis* [3, 14, 15]. This different outcome could be due to the difference in the nature of the stem structures between snapdragon and gerberas and *Calendula officinalis*. The prevention of stem bending by ethephon or enhancement of stem bending by STS observed in this study was consistent with the work of Naing et al. [2], who recently reported the preventive role of ethephon in stem bending and the stimulatory role of STS in stem bending in other snapdragon cultivars. However, our results were not consistent with the results of previous studies that reported the inhibition of stem curvature by STS or 1-MCP [6, 7]. In addition, our results differed from those of Celikel et al. [5], who suggested that ethylene inhibitors did not significantly affect the gravitropic curvature and bending of snapdragon.

In addition to the curvature degree, RSE also affected stem bending; the RSE results of all treatments observed in part II (STS > control > SNP > ethephon) were correlated with bending rates. In addition, the RSE observed in part II of bending stems was obviously longer than that of unbending stems, unlike in part I (Fig. 3). The RSE in part III was relatively lower compared to that in the other parts (II and I); thus, it would not affect bending. Inhibition of stem elongation in cut flowers by ethephon [2, 9, 11] and delaying stem bending by its application in vase life solution have been reported in previous studies [2, 11, 16]. In this study, higher reduction of bending rate by SNP over control at 6 DAT was associated with a significant reduction of RSE in part II. Previous studies have reported the role of SNPs in delaying stem bending in gerbera and *Calendula officinalis* L. [3, 14, 15]; however, they did not investigate the role of SNPs in the inhibition of RSE. Our results suggest that the delaying stem of bending using SNP observed in previous studies could partly be also explained by its inhibitory effect on RSE.

In this study, the important role of lignin content in stem bending was observed, whereas the presence of slightly higher content in the treatments caused delaying of the bending rates at 6 and 9 DAT. In addition, the increase in bending rates along with a longer vase life period was linked to continuous decreases in lignin content. Similarly, previous studies have reported that the stem bending of cut flowers is associated with poor lignification mechanism [2–4, 17]. Naing et al. [3] claimed that SNP could maintain lignin content by triggering lignin biosynthetic genes. In this study, SNP did not significantly promote lignin content compared to the control, while ethephon significantly promoted it. The role of ethephon in lignin accumulation via triggering lignin biosynthetic genes (*PAL* and *4CL*) and improvement of resistance to stem breakage has been reported in many plant species [13, 18–23]. Therefore, the higher lignin content in the ethephon-treated stems than in other treatments could be due to the accumulation of higher ethylene in the former than in the latter. As expected, there were significant or slight associations between ethylene production and lignin content in the treatments. The higher ethylene production in the control than the STS treatment supported the presence of higher lignin content in the stems of the former. The expression levels of *PAL* and *4CL* in ethephon, STS, and control were correlated with lignin content. *ACO* and *ACS* genes are involved in ethylene biosynthesis in plants, as well as other cellular processes such as cell wall and membrane metabolisms, by switching on signal transduction on ethylene [24, 25]. In this study, expression of ACO2 was associated with ethylene production at 6 DAT. Therefore, expression of *ACO2*, *PAL,* and *4CL* at 6 DAT also supported the association between ethylene and lignin production. The maintenance of lignin content at lower levels by SNP than ethephon could be due to the inhibitory effect of SNP on ethylene production, which has been reported in carnations [26].

Unfortunately, ethephon rapidly accelerated petal senescence due to its higher ethylene production, which strongly induces petal senescence and abscission. In contrast, STS significantly reduced petal senescence owing to its inhibitory effect on ethylene production. The role of ethylene in petal senescence and abscission has been reported in various flowers [27, 28]. Various supplements (such as BA, GA_3_, CaCl_2_, and sugar) have been used to delay stem bending and provide better postharvest quality performance in cut tulip flowers [11, 29]. Moreover, the role of CaCl_2_ in delaying stem bending in gerbera cut flowers has been previously reported [30, 31]. It is likely that CaCl_2_ maintains cell wall rigidity by binding to pectin molecules, thereby increasing cell wall stiffness that might result in delayed bending [32, 33]. Moreover, calcium has been reported to reduce the rate of plant senescence [34]. In this study, addition of the supplements (BA, GA_3_, and CaCl_2_) did not significantly reduce petal senescence in the control, STS, and SNP; however, the supplements relieved the negative effect of ethephon on senescence (from 90 to 50%) at 9 DAT, as also observed in the study done van Doorn et al. [11]. Another advantage of the supplements was that they reduced the bending rates of the control, STS, and SNP treatments. In summary, at 9 DAT, ethephon completely prevented stem bending of snapdragon cut flowers whereas STS significantly stimulated it. SNP did not significantly affect stem bending compared to the control. Generally, stem bending is linked to a higher degree of curvature and RSE as well as lower ethylene production and lignin content. The combined use of ethephon and supplements can prevent stem bending with a lower rate of petal senescence and is therefore recommended to be used by growers and in the floricultural industry.

## Conclusion

This study demonstrated the effects of ethephon, STS, and SNP on the stem bending of cut snapdragon flowers. Ethephon was found to completely prevent bending within the study period (9 days). Moreover, the bending rate of STS was higher than that of the control and SNP did not significantly delay bending compared to the control, particularly at 9 DAT. Stem bending was correlated with degree of stem curvature, RSE, ethylene production, and lignin content and this was proven by expression analysis of genes involved in the ethylene and lignin biosynthetic pathways. The combined use of supplements also delayed stem bending and petal senescence. Ethephon most effectively delayed stem bending of snapdragon and combining with supplements relieved approximately half of the ethephon-induced petal senescence. Therefore, we expect that the information obtained from this study would be valuable for prolonging the appearance of cut flowers that face early stem bending problems.

## Materials and methods

### Plant material

Cut snapdragon flowers (*Antirrhinum majus* L. Busasol) were obtained from a farm in Busan. Flowers with 4–6 open flowers per inflorescence and free of mechanical and disease defects were selected. These selected flowers were harvested in the early morning and immediately transported to the laboratory of Kyungpook National University, a journey of 1–2 h. The flowers were horizontally placed in flower box during transportation. After arriving at the laboratory, the flower stems were trimmed to a length of 50 cm and excess leaves were gently removed.

### Treating cut flowers with STS, ethephon, and SNP alone or in combination with supplements

Prior to treating the cut flowers, a stock solution of silver thiosulfate (STS) was prepared by adding 0.2 mM silver nitrate (Sigma Aldrich) to 1.2 mM sodium thiosulphate (Sigma Aldrich). The cut flowers were separately placed in bottles (1 L) containing 0.2 mM of STS, 67 mM of sodium nitroprusside (SNP; Sigma Aldrich), and 0.25 mM of 2-chloroethylphosphonic acid (ethephon; Sigma Aldrich), with or without supplements [0.75 mM of Gibberellic acid 3 (GA_3_; Duchefa Biochemie) + 0.25 mM of benzyladenine (BA; Duchefa Biochemie) + 10 mM of calcium chloride (CaCl_2;_ Duchefa Biochemie)]. Flowers placed in bottles containing only distilled water were used as controls. One treatment contained three bottles with ten flowers per bottle. The treatment concentration ranges were determined based on previous studies [11, 26]. After treatment for 24 h, the stems were thoroughly washed with tap water and placed into new bottles containing only distilled water (500 mL). The bottles were then maintained in a controlled chamber at a temperature of 20 °C, relative humidity of 60%, light intensity of 15 µmol m^­2^ s^−1^, and photoperiod of 12 h.

### Measurement of angle of curvature (degree)

**Fig. 5:**
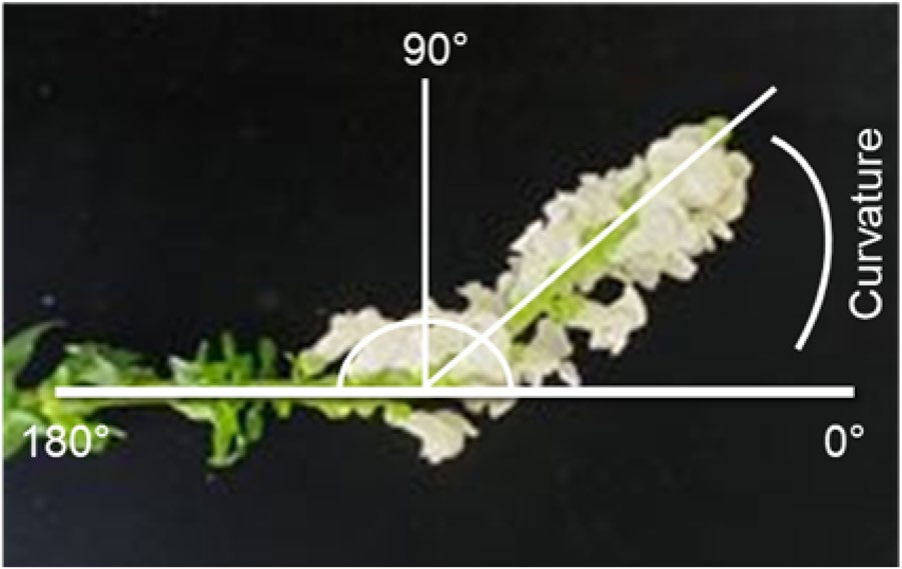
Illustration of the angle of curvature (degree) of snapdragon spike

Induction of the stem curvature is the first phase of stem bending. In this study, we investigated whether ethephon, STS, and SNP could control the degree of stem curvature of snapdragon cut flowers. Stem curvature was measured using a protractor and described in degrees (Fig. 5). A stem curvature of 0° was defined as a straight stem. The stem gets closer to bending as the curvature gets closer to 180°. The upper part of the stem with an angle of more than 45° was considered as a bent stem, as a stem with a curvature of more than 45° is not fit for commercial purposes. The curvature of all stems (30) was measured on days 3, 6, and 9 after treatment (DAT). Each measurement contained ten flower stems and was performed three times.

### Bent stem (% of total)

The stems that exhibited degrees of curvature greater than 45° were considered to be bent stems. The bent stems were counted at 3, 6, and 9 DAT and the results were described as percentages.

### Relative shoot elongation (SRE) (millimeter)

**Fig. 6:**
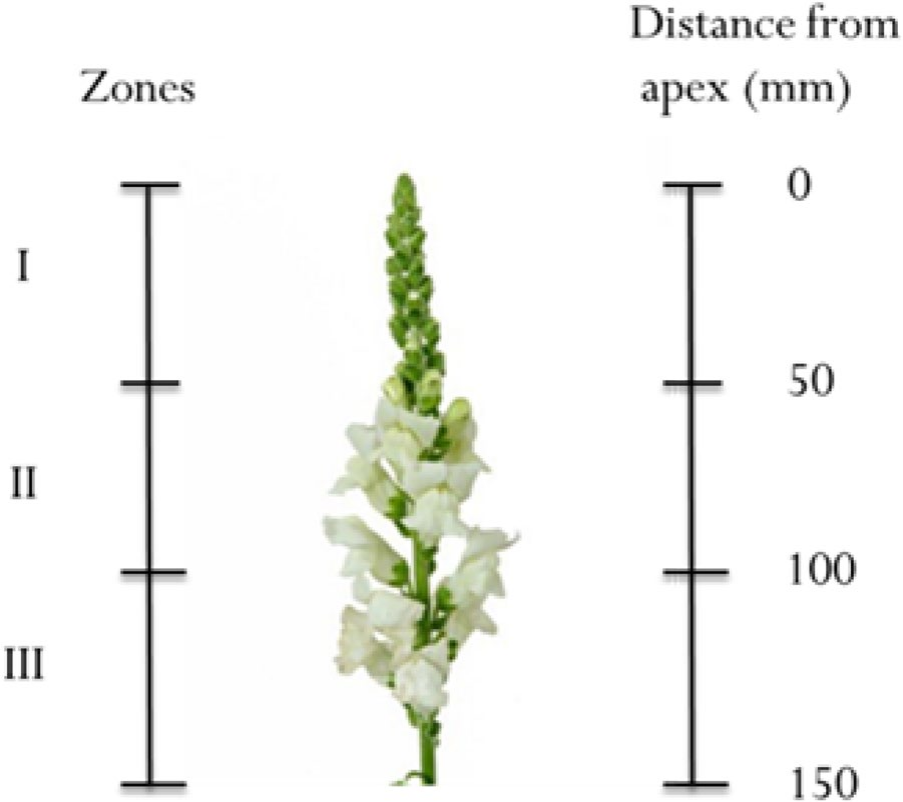
Illustration of the dividing of the three different parts of shoots for investigation of relative shoot elongation of snapdragon cut flowers

To clarify whether stem bending was associated with RSE, elongation was assessed. Briefly, the shoots were divided into three zones: zones I, II, and III starting from the apex of the inflorescence stem at 50 mm intervals. The zones were marked with a felt pen before pulsing with chemical treatment (Fig. 6). The elongated length was monitored at 6 DAT and described in millimeters (mm). Each treatment contained ten flowers and the measurements were repeated three times.

### Analysis of lignin content

Lignin analysis was performed according to the method described by Syros et al. [35]: stem parts from the bending zone, which were sampled at 3, 6, and 9 DAT, were pulverized in 95% ethanol using a mortar and pestle. Then, the homogenates were centrifuged at 1000*g* for 10 min. The pellets were washed with 95% ethanol and 95% ethanol: *n*-hexane solution (1:2 v/v) three times. After drying, 20 mg of the deposits were dissolved in 0.5 mL of 25% acetyl bromide in ice acetic acid for 30 min at 70 °C. After rapid cooling, 0.9 mL NaOH (2 M) was added to stop the reaction, which was followed by the addition of 0.1 mL hydroxylamine-HCl (7.5 M) and 5 mL ice acetic acid. Then, 0.1 mL of reaction mixture was diluted with 3 mL of ice-cold acetic acid. After centrifugation at 1000×*g* for 5 min, the supernatant was measured at 280 nm. Lignin content was expressed as a 280 mg^−1^ protein. Three independent bending zones were used.

### Measurement of ethylene production

Ethylene production was analyzed from the bending zones of stems treated with STS, ethephon, and control. The bending zones were sampled from 6 DAT. Samples of approximately 2–3 g were placed in 30 mL Erlenmeyer flasks, sealed with rubber serum caps, and incubated for 20 h at room temperature for ethylene accumulation. Ethylene production was measured using gas chromatography (7820 GC) and the ethylene production rates were calculated and expressed as described by Xu et al. [36]. Three independent bending zones were used for each analysis and analysis was performed three times.

### Expressional analysis of genes involved in lignin and ethylene biosynthesis

Total RNA was extracted from 100 mg stem segments from the bending zones, which were sampled at 3, 6, and 9 DAT using the RNeasy Plant Mini Kit (Qiagen, Hilden, Germany). The cDNA was synthesized from 1 µg of total RNA using an oligo dT20 primer and a reverse transcription kit (ReverTra Ace-á, Toyobo, Japan). The mRNA levels of the lignin biosynthetic genes (*PAL* (DQ866660.1) and *4CL* (Y15607.1)) were analyzed at 3, 6, and 9 DAT, but those of ethylene biosynthetic genes (*ACS1* (AF083814.2), *ACO1* (AY333925.1), and *ACO2* (AY333926.1)) were analyzed only at 6 DAT, using the StepOnePlus Real-Time PCR system (Thermo Fisher Scientific, Waltham, MA, United States). Relative gene expression was calculated using the quantitative-comparative method *C*_T_ (ΔΔ*C*_T_). The primers and PCR conditions used for this analysis were listed in Additional file 1: Table S1. Three independent bending zones were used for each analysis and analysis was performed three times.

### Petal senescence

Petal senescence was assessed by observing wilting and browning at the corners of the petals. When more than 50% of the petals from the inflorescence showed senescing symptoms, the flowers were considered to be senescing. Senescence was evaluated in all flowers at 3, 6, and 9 DAT, and the data were expressed as parentages.

### Statistical analysis

Data were analyzed using SPSS v. 11.09 (IBM Corporation, Armonk, NY, USA) and presented as the mean of the three replicates. The least significant difference test was used to assess the differences between mean values. The significance level was set at a P-value < 0.05.

## Supplementary Information

**Additional file 1: Table S1**. Primer sequences used for the detection of genes related to ethylene biosynthesis and lignin biosynthesis by qRT-PCR.

## Data Availability

All datasets used in this study are available from the corresponding author on reasonable request.

## Abbreviations

*4CL*: 4-coumarate: CoA ligase
ACS1: 1-Aminocyclopropane-1-carboxylic acid synthetase 1
ACO1: 1-Aminocyclopropane-1-carboxylic acid oxidase 1
ACO2: 1-Aminocyclopropane-1-carboxylic acid oxidase 2
BA: 6-Benzyladenine
CaCl_2_: Calcium chloride
GA_3_: Gibberellic acid 3
*PAL*: Phenylalanine ammonia-lyase
SNP: Sodium nitroprusside
STS: Silver thiosulfate
